# Echinacoside inhibits PASMCs calcium overload to prevent hypoxic pulmonary artery remodeling by regulating TRPC1/4/6 and calmodulin

**DOI:** 10.1515/med-2024-1044

**Published:** 2024-10-04

**Authors:** Enqi Zhao, Jinyu Wang, Yuefu Zhao, Qingqing Xia, Hongmai Wang, Zhanqiang Li, Cen Li, Xiangyun Gai

**Affiliations:** School of Pharmacy, Qinghai Minzu University, Xining, Qinghai, 810007, China; Qinghai University Plateau Medicine Research Center, Xining, Qinghai Province, China; Northwest Institute of Plateau Biology, Chinese Academy of Sciences, Xining, Qinghai Province, China

**Keywords:** pulmonary hypertension, echinacoside, hypoxic, calcium, pulmonary vascular remodeling

## Abstract

**Abstract:**

Research indicates that hypoxic pulmonary hypertension (HPH) potentially stimulates the sympathetic nervous system, which may increase norepinephrine (NE) release and cause excessive Ca^2+^ influx into pulmonary artery smooth muscle cells (PASMCs), leading to calcium overload and abnormal PASMC proliferation, factors closely associated with pulmonary vascular remodeling (PVR). This study investigates the potential mechanisms underlying echinacoside (ECH) treatment in HPH.

**Method:**

In the *in vitro* experiment, NE-induced PASMCs were used to simulate HPH-induced PASMCs’ calcium overload and abnormal proliferation. Postincubation with ECH, [Ca^2+^]_cyt_ changes were detected using Fluo-4 AM. Flow cytometry was employed to ascertain ECH’s inhibitory effect on PASMCs proliferation. For *in vivo* experiments, rats were exposed to a hypoxic and low-pressure oxygen environment to establish the HPH model. Post-ECH treatment, hematoxylin and eosin (HE) staining was conducted to assess PVR, and western blot analysis was used to examine protein expression in the lung tissues of the different groups.

**Results:**

ECH was observed to inhibit [Ca^2+^]_cyt_ increase in NE-induced PASMCs in a concentration-dependent manner, effectively reducing abnormal cell proliferation. It also reduced the expression of Transient receptor potential channel (TRPC) 1 (TRPC1), TRPC4, TRPC6, and calmodulin in PASMCs. *In vivo* studies demonstrated that ECH lowered the expression of these proteins in lung tissues of HPH rats, significantly decreased mean pulmonary artery pressure, and mitigated PVR.

## 1 Introduction

Hypoxic pulmonary hypertension (HPH) is a progressive syndrome resulting from prolonged hypoxia, characterized by hypoxic pulmonary vascular contraction and hypoxic pulmonary vascular remodeling (PVR). These changes cause a continual rise in mean pulmonary artery pressure (mPAP), leading to right heart hypertrophy, and potentially resulting in right heart failure and death [1]. Elevated cytoplasmic Ca^2+^ concentration ([Ca^2+^]_cyt_) in pulmonary artery smooth muscle cells (PASMCs) is a key factor in both pulmonary vasoconstriction and vascular remodeling [2,3]. Vasoactive substances regulate pulmonary artery blood pressure by activating G protein-coupled phospholipase-C (PLC), which catalyzes the conversion of PLC into inositol triphosphate and diacylglycerol. This activation triggers the opening of receptor-activated Ca^2+^ channels (ROCCs) and stimulates inositol triphosphate (IP3), which reduces intracellular Ca^2+^ and activates store-operated calcium channels (SOCCs)[4]. The activation of both ROCC and SOCC facilitates the influx of extracellular Ca^2+^, elevating intracellular [Ca^2+^]_cyt_ [5]. Transient receptor potential channels (TRPCs), specifically TRPC1, TRPC4, and TRPC6, are major components of SOCC and ROCC and play a crucial role in pulmonary vascular function [6,7,8]. Furthermore, calmodulin (CaM), a primary intracellular Ca^2+^ sensor, is ubiquitous in eukaryotic cells and regulates numerous signaling pathways, including those involved in growth and proliferation [9]. An increase in intracellular [Ca^2+^]_cyt_ activates CaM, forming Ca^2+^/CaM complexes critical in vascular smooth muscle contraction [10].

**Figure 1:**
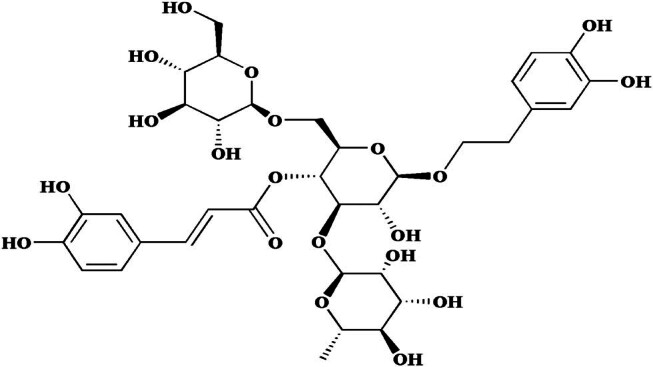
Chemical structure of ECH.

Echinacoside (ECH) is a phenethyl alcohol glycoside compound (Figure 1), predominantly extracted from *Cistanche tubulosa* (Schenk) Wight. Our previous study has demonstrated that ECH exhibits a concentration-dependent relaxing effect on the rat pulmonary artery ring *in vitro*, potentially attributable to its inhibition of Ca^2+^ inflow and release in PASMCs [11]. In addition, ECH has been shown to ameliorate PVR in HPH rats, decrease mPAP, and offer therapeutic benefits in the management of HPH [12]. However, the precise mechanisms and targets of ECH’s action in HPH remain to be elucidated. Consequently, this study aims to preliminarily investigate the underlying mechanisms of ECH’s preventative and therapeutic effects on HPH.

## 2 Materials and methods

### 2.1 Chemicals and reagents

ECH (BWC9053-2016, HPLC ≥ 98%) was purchased from Henan North Weiye Metrology Technology Co., Ltd. SolarBio Science & Technology Co., Ltd. (Beijing, China) supplied dimethylsulfoxide (DMSO) and trypsin. Shanghai Yuanye provided the noradrenaline (NE ≥ 99%). Gibco BRL Co., Ltd (Gaithersburg, MD, USA) delivered high glucose Dulbecco’s modified Eagle’s medium (DMEM) and fetal bovine serum (FBS). α-Smooth muscle actin (α-SMA Rabbit mAb (A17910) was supplied from ABclonal Biotech Co., Ltd (Wuhan, China). Proliferating cell nuclear antigen (PCNA) polyclonal antibody (10205-2-AP) was supplied from Wuhan Sanying Biotechnology Co., Ltd (Wuhan, China). 4′,6-diamidino-2-phenylindole (DAPI) dyeing reagent (G1012) was purchased from Wuhan Xavier Biotechnology Co., Ltd (Wuhan, China). Two-step plus Poly-HRP anti-mouse/rabbit IgG detection system (E-IR-R217) was from Elabscince Biotechnology Co., Ltd (Wuhan, China). Fluo-4, AM, cell-permeant (F14217), and TRPC4 (PA5-95508) were acquired from Invitrogen Corp (Carlsbad, USA). Rabbit monoclonal antibodies to Calmodulin1/2/3-C-terminal (ab124742), TRPC1 (ab192031), and TRPC6 (ab101622) were obtained from Abcam Corp (Cambridge, MA). Goat anti-rabbit secondary (AS014) was purchased from ABclonal Technology Co., Ltd. (Wuhan, China). Beyotime Biotechnology Co., Ltd (Shanghai, China) provided the improved bicinchoninic acid assay (BCA) protein test kit. Thermo Fisher Scientific Co., Ltd. (Shanghai, China) delivered the protein molecular weight marker. Amersham International (Amersham, UK) contributed to the enhanced chemiluminescence (ECL) reagents.

### 2.2 Experimental animals

Male Sprague Dawley rats weighing 180–250 g at 7 weeks of age were purchased from the Shanxi Experimental Animal Center (License number: SCXK 2018-001). The Ethics Committee on Laboratory Animals of Qinghai Minzu University approved all animal experiment procedures.

### 2.3 *In vitro* experiment

#### 2.3.1 Isolation of rat PASMCs

Pentobarbital sodium (40–60 mg/kg) was administered intraperitoneally to anesthetize the rats. Then, for the next 5 min, they were immersed in a 75% ethanol solution. One minute after anesthesia, lung tissue was harvested; two to three distal small pulmonary arteries, endothelial cells, and outer membrane cells were removed. The middle layer, which had been separated, was sectioned into small tissue blocks measuring 1 mm × 1 mm × 1 mm. These blocks were promptly placed at the bottom of a 25 T culture flask, with the flask positioned in an inverted manner. Subsequently, a culture medium consisting of DMEM and 20% FBS was introduced into the flask. The cells were cultured at 37°C in a humidified atmosphere with 5% CO_2_ for approximately 2 h. During this time, the small tissue blocks adhered to the inner surface of the flask. After that, the inverted flasks were placed in the incubator for further incubation. Upon reaching 80% confluency, the cells underwent digestion using a 0.25% trypsin solution. Subsequently, they were transferred into culture bottles containing high-glucose DMEM with penicillin (100 U/mL), streptomycin (100 U/mL), and 15% FBS. The passaging process was then carried out, maintaining a ratio of 1:3. All tests employed cells range from three to eight passages.

#### 2.3.2 Identification of PASMCs

When the cells attained 80% confluence, they were plated onto coverslips for immunohistochemical and immunofluorescence examination. The cells were treated with a 10% paraformaldehyde solution for 20 min to fix them. Subsequently, a 0.4% Triton X-100 solution was applied to the cells for 20 min to induce permeabilization. A 3% hydrogen peroxide (H_2_O_2_) solution was introduced at ambient temperature for 10 min to deactivate the naturally occurring enzymes. The samples were blocked with normal goat serum at 37℃ for 30 min. Subsequently, an adequate dilution of α-SMA monoclonal antibody (primary antibody at a ratio of 1:50) was added to the samples, which were then incubated overnight at 4°C. Following three PBS washes, the cells were put in a diaminobenzidine working solution and treated for 30 min at 37°C with a secondary antibody. The positive signal was brown-yellow or brown observed under the microscope. After slices were washed in distilled water, they were redyed, dehydrated, transparent, and sealed.

#### 2.3.3 CCK-8 assay for cell survival analyses

The assessment of cell viability was conducted using CCK8 analysis. The 96-well plates were seeded with a volume of 200 μL of the medium, containing a density of 2,000 cells per well. After that, the plates were placed in an incubator that was set to 37°C, 5% CO_2_, and 100% humidity, and left undisturbed for 24 h. Once the cells had completely attached to the surface of the wall, a serum-free media was cultured for 24 h to synchronize the cells. Cells in each well were then stimulated with 10 µL of different drugs. The 96-well plates underwent incubation for 24 h at 37°C within a cell incubator that maintained a 5% CO_2_ concentration and 100% humidity. A microplate reader was employed to determine absorbance at 450 nm.

#### 2.3.4 Flow cytometry for cell cycle distribution

Flow cytometry was employed to assess the cell distribution in the S, G2/M, and G0/G1 phases. PASMCs were seeded into six-well plates, and after the cells were exposed to the drug, they were digested by trypsin, collected, centrifuged, and rinsed twice with cold PBS (1 mL). Cells were resuspended, immobilized in 70% cold ethanol, and placed overnight at −20°C. After centrifuging the immobilized cells, aspirating the supernatant, adding 1 mL of PBS, and resuspending the cells, they remained for 15 min at room temperature. After centrifugation and aspiration of the supernatant solution, 100 µL RNaseA was introduced to suspend the cells entirely, the propidium iodide solution was added and mixed thoroughly, a 300 mesh sieve was used to filter the cells, and red fluorescence at 488 nm was detected by flow cytometry.

#### 2.3.5 Ca^2+^ measurements in rat PASMCs

Glass coverslips were used to seed the cells in six-well plates. The cells were cultivated in a serum-free DMEM medium for 24 h once the cell density reached 80%. Cytoplasmic ratiometric calcium probe Fluo-4 AM (5 µM) was loaded in a 5% CO_2_, 37°C incubator for 30 min. Hanks’ balanced salt solution was employed to wash away the residual color. Fluo-4 AM-loaded cells were cultured on a live cell workstation in a whole medium. Cells loaded with Fluo-4 AM were observed with a confocal laser microscope at 488 nm for 15 s, and data were recorded in real time for 15 min. Using Image Pro-Plus 6.0 software, intracellular calcium concentrations were determined by measuring the average fluorescence intensity.

### 2.4 *In vivo* experiment

#### 2.4.1 Modeling and treatment of rats

Four groups of rats were randomly assigned: (1) normoxic group, (2) HPH group, (3) HPH + ECH (15 mg/kg) group, and (4) HPH + ECH (30 mg/kg) group. In each group, there were eight rats. The rats in all the groups, except for group (1), were kept in an environment of 22 ± 2℃ for 28 days, with food provided in a hypoxia low-pressure oxygen chamber (DY3000, Guizhou, China) at 5,000 m simulated altitude. The bedding was changed every 3 days, and the animals were kept in a 12-h cycle of light and dark. The HPH model was established in rats. Commencing from the first day of the model establishment, rats in groups 3 and 4 received intragastric ECH therapy at corresponding dosages. Concurrently, rats in groups 1 and 2 were administered an equivalent volume of normal saline daily. This regimen was maintained for 28 days.

#### 2.4.2 mPAP measurement

Pentobarbital sodium (40–60 mg/kg) was administered intraperitoneally to anesthetize the rats after 28 days of Modeling. A silicone catheter (outside diameter 0.9 mm) was inserted intravenously through the right ventricle (RV) and tricuspid valve into the pulmonary artery from the right external jugular vein. The MP160 pressure signal acquisition system (Biopac, California, USA) was used to record mPAP while intubating.

#### 2.4.3 Right heart hypertrophy index

The RV and heart were separated when the chest was opened. By using the RV’s wet weight ratio to the left ventricle’s (LV) plus septum (SP), the right ventricular hypertrophy index was computed through the following formula: RV/(LV + SP) is the right heart hypertrophy index (RVHI).

#### 2.4.4 Pathological detection and analysis of lung tissue in rats

Following their embedding in paraffin wax and cutting into 4 μm thick slices, the lung tissues from each group of rats preserved in 4% paraformaldehyde were hydrated with graded ethanol, dewaxed in xylene, and stained with H&E stain. Meanwhile, the expression of α-SMA (1:400) and PCNA (1:400) in lung tissues of rats in each group was observed by immunofluorescence stainin. The slices were imaged using a Panoramic 250 digital biopsy scanner manufactured by 3DHISTECH (Hungary). The pulmonary artery diameter, wall area, wall thickness (WT), total wall area, lumen area, and number of positive co-expressing cells stained by immunofluorescenc were all determined using Image-pro Plus 6.0 software. The indices of pulmonary vascular morphology, including pulmonary vascular wall thickness/outer diameter (WT%), pulmonary vascular lumen area/total wall area (LA%), and pulmonary vascular wall area/total wall area (WA%), were computed to facilitate subsequent statistical analysis.

#### 2.4.5 Western blotting analysis

A precise measurement of 100 mg of lung tissue is obtained, followed by the subsequent tissue division. Based on the established tissue weight-to-PBS ratio of 1:9, the precooled PBS should be added to the sample and homogenized meticulously while maintaining a low temperature on ice. After the homogenate was transferred to a 1.5 mL centrifuge tube, it was centrifuged at 4°C, 12,000 rpm, for 15 min. The liquid portion of the sample was gathered, carefully wrapped, and afterward kept at −80°C. This was done to facilitate the subsequent protein quantification and electrophoresis tests. The packed protein was acquired and measured using the BCA protein quantification technique. SDS-PAGE was employed to separate protein bands of equal quantity from the protein lysate, which were deposited onto a nitrocellulose membrane. The membrane should be sealed using a 5% skimmed milk tris-buffered saline (TBS) solution for 1 h. The enclosed membrane was subjected to an overnight incubation at 4°C, during which it was exposed to primary antibodies including anti-beta-actin (1:400), anti-CaM (1:1,000), anti-TRPC1 (1:1,000), anti-TRPC4 (1:1,000), and anti-TRPC6 (1:1,000). The nitrocellulose membrane undergoes a washing process using TBS-Tween. The goat anti-mouse and anti-rabbit secondary antibodies (1:500) were combined with the membrane and horseradish peroxidase, followed by incubation at ambient temperature for 1 h. Using the ECL reagent, the immune response’s banding signal was detected. An imaging technique was employed to measure the strength of a single strip in the western blot. Comparing each protein’s strength to that of β-actin yields its relative strength.

**Figure 2:**
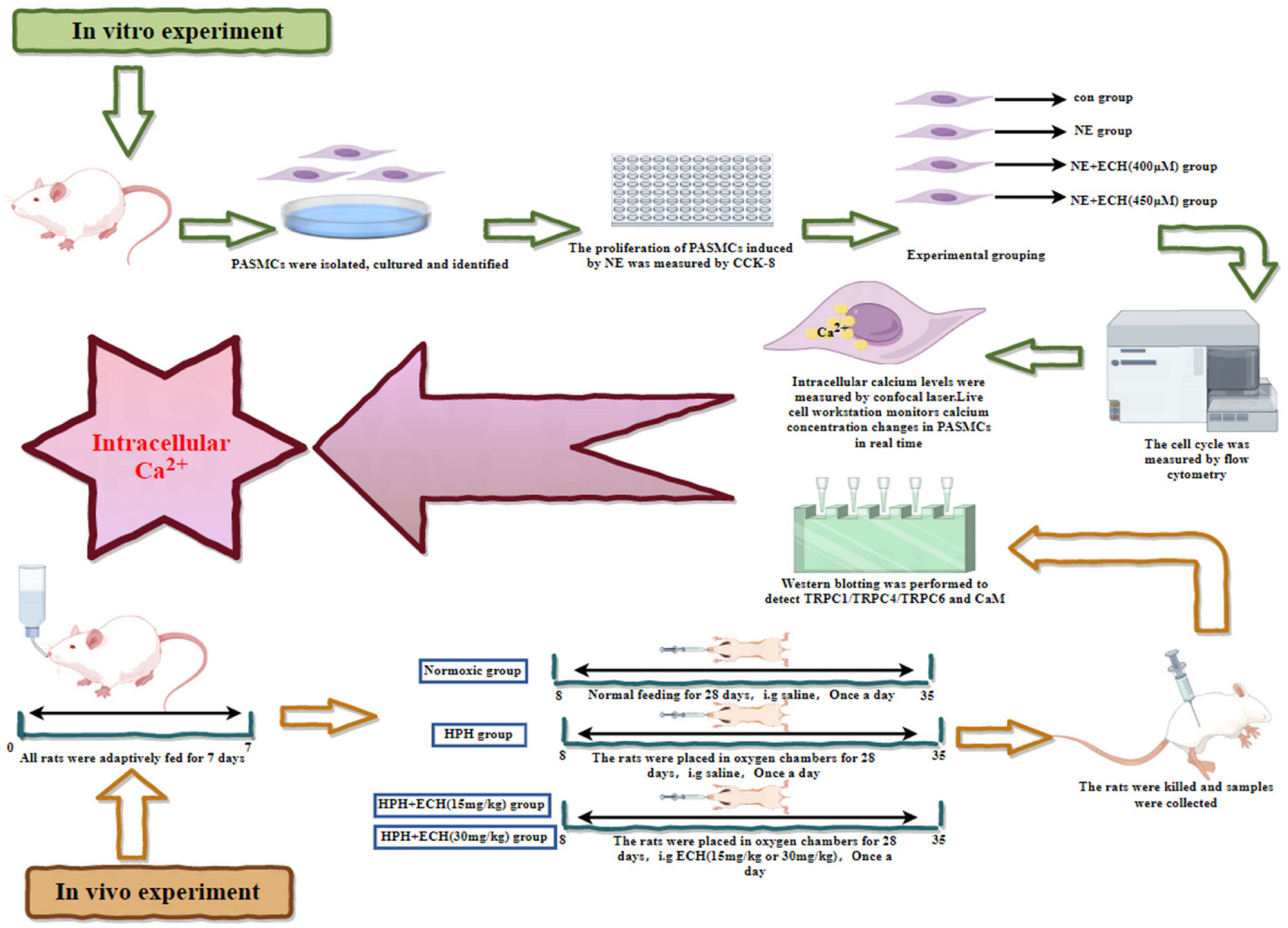
General flowchart of experiment.

The flowchart of this experiment is shown in Figure 2.

### 2.5 Statistical analysis

The outcomes are presented as mean ± standard deviation. The statistical methodology employed in this study included one-way analysis of variance, LSD *post hoc* test, and multiple comparisons. *P* < 0.05 represents statistical significance.

## 3 Results

### 3.1 Extraction and identification of primary rat PASMCs

**Figure 3:**
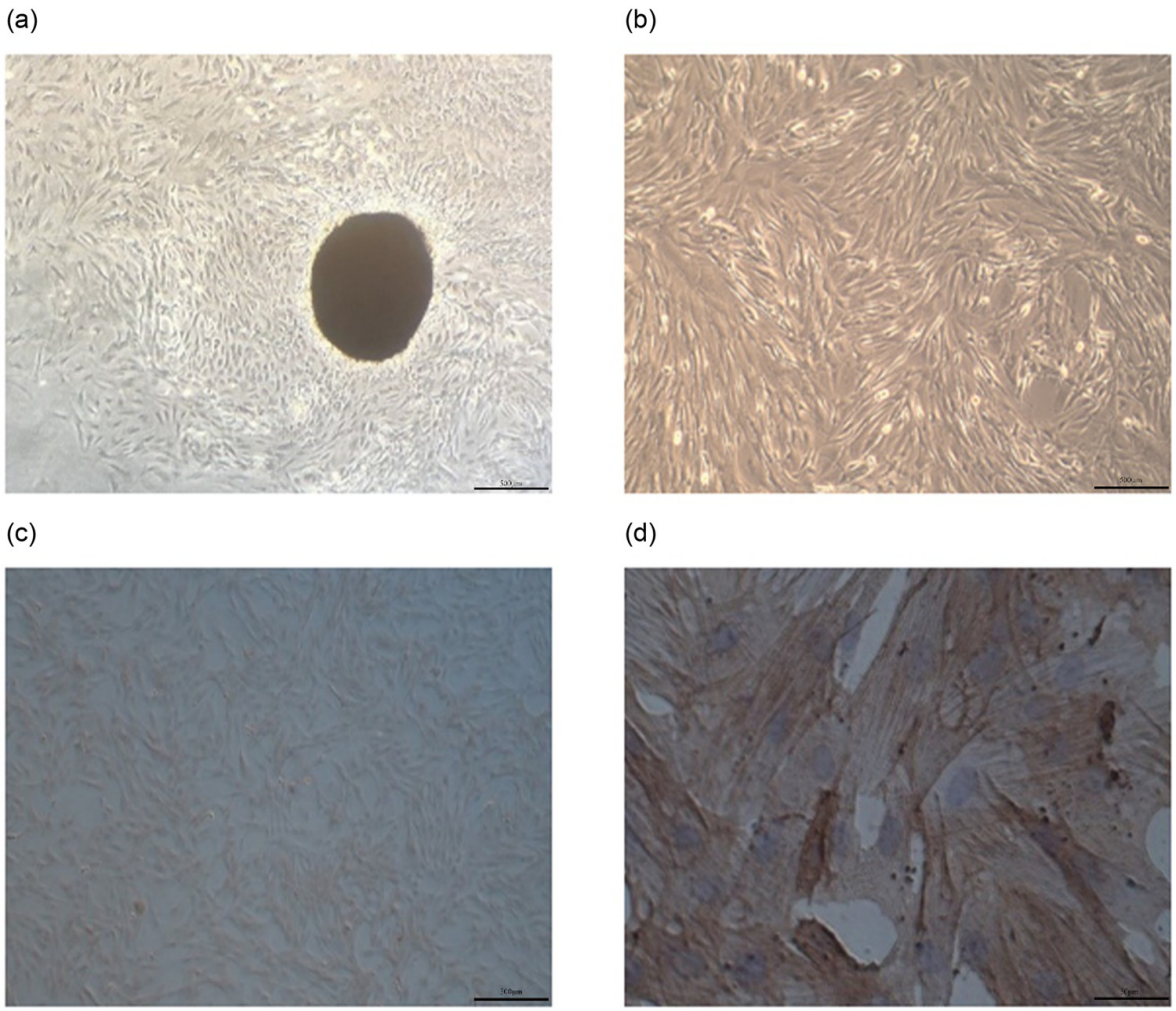
Cultured rat PASMCs under an inverted microscope and the chemical structure of ECH. (a) First-generation PASMCs were examined at 40× through an inverted microscope. (b) Inverted microscopic observation of first-generation PASMCs (40×). (c) PASMCs, after immunohistochemical staining, were observed under an inverted microscope (40×), showing a yellow positive signal (PASMC purity >95%). (d) PASMCs after immunohistochemical staining were observed under an inverted microscope (400×), and myofibrils stained brown were observed in the cells.

Under an inverted phase-contrast microscope, after 4–5 days of attachment of the tissue block to the wall, cells can be observed crawling out from around it and forming a radial array of cells of different sizes and shapes (Figure 3a). When these cells are passed on to the first generation, the cells are observed to have a regular morphology and a long spindle shape. At higher densities, the cells grow in clusters (Figure 3b). At low magnification, most cells were “peak-valley” shaped. The PASMCs were brownish-yellow by immunohistochemical staining (PASMCs purity was >95%) (Figure 3c). Brown-stained myofilaments, or smooth muscle cells (SMCs) expressing α-SMA, have been observed in the cytoplasm at a greater magnification (Figure 3d). In cells without primary antibodies, no brown myofibrillar proteins were observed (Figure 3b). Within eight generations, good morphological structure and function were maintained.

### 3.2 Various levels of NE increased PASMCs proliferation

**Figure 4:**
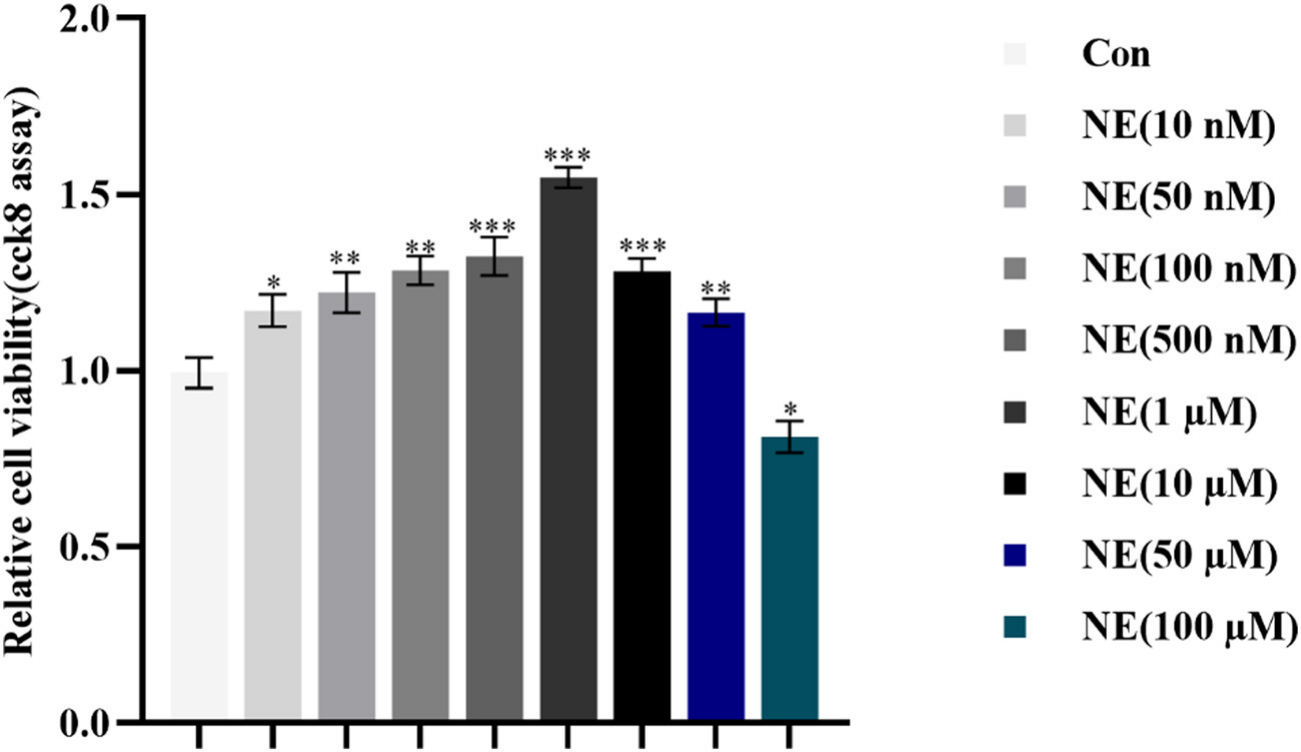
Cell viability was measured with CCK8 to screen for optimal concentration of PASMC proliferation by NE. ***p < 0.001, **p < 0.01, *p < 0.05, against control group. “Con” means control.

As demonstrated in Figure 4, NE stimulates PASMC proliferation in the range of 10 nM to 50 µM, as measured by an increase in cell viability relative to the control group (*p* < 0.05). The NE (1 µM) group showed the highest significance (*p* < 0.001) when compared to the control group, suggesting that PASMC proliferation peaked at 1 µM NE concentrations. Therefore, PH-induced sympathetic nervous system activation, pulmonary vasoconstriction, and PASMC proliferation all contribute to the elevated NE release. Since this proliferative effect was most pronounced at 1 μM, NE was set at 1 μM in subsequent experiments.

### 3.3 Impact of ECH on NE-induced PASMCs proliferation

**Figure 5:**
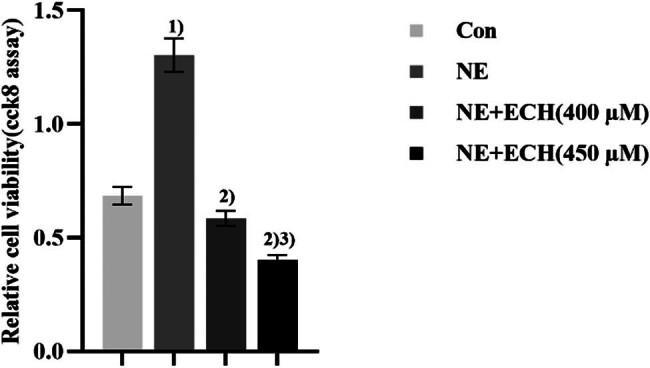
The effect of ECH on PASMCs proliferation produced by NE. ^1)^ *p* < 0.01 vs control group; ^2)^ *p* < 0.01 vs NE group; ^3)^ *p* < 0.01 vs NE + ECH (400 µM) group. “Con” stands for “control” (*n* = 6).

Figure 5 shows that in comparison to the control group, cell viability was significantly higher in the NE group (*p* < 0.01), demonstrating the efficacy of the NE modeling approach. In contrast to the NE group, the cell viability of the NE + ECH (400 μM) group exhibited a substantial decrease (*p* < 0.05). This finding proves that preincubation with ECH (400 μM) effectively suppresses the NE-induced proliferation of PASMCs. In addition, it was observed that the cell viability of the NE + ECH (450 μM) group exhibited a statistically significant decrease compared to the NE + ECH (400 μM) group. The findings of this study suggest that ECH exhibits a concentration-dependent inhibition of the proliferation of PASMCs triggered by NE (*p* < 0.01).

### 3.4 Distribution of the cell cycle using flow cytometry analysis

**Figure 6:**
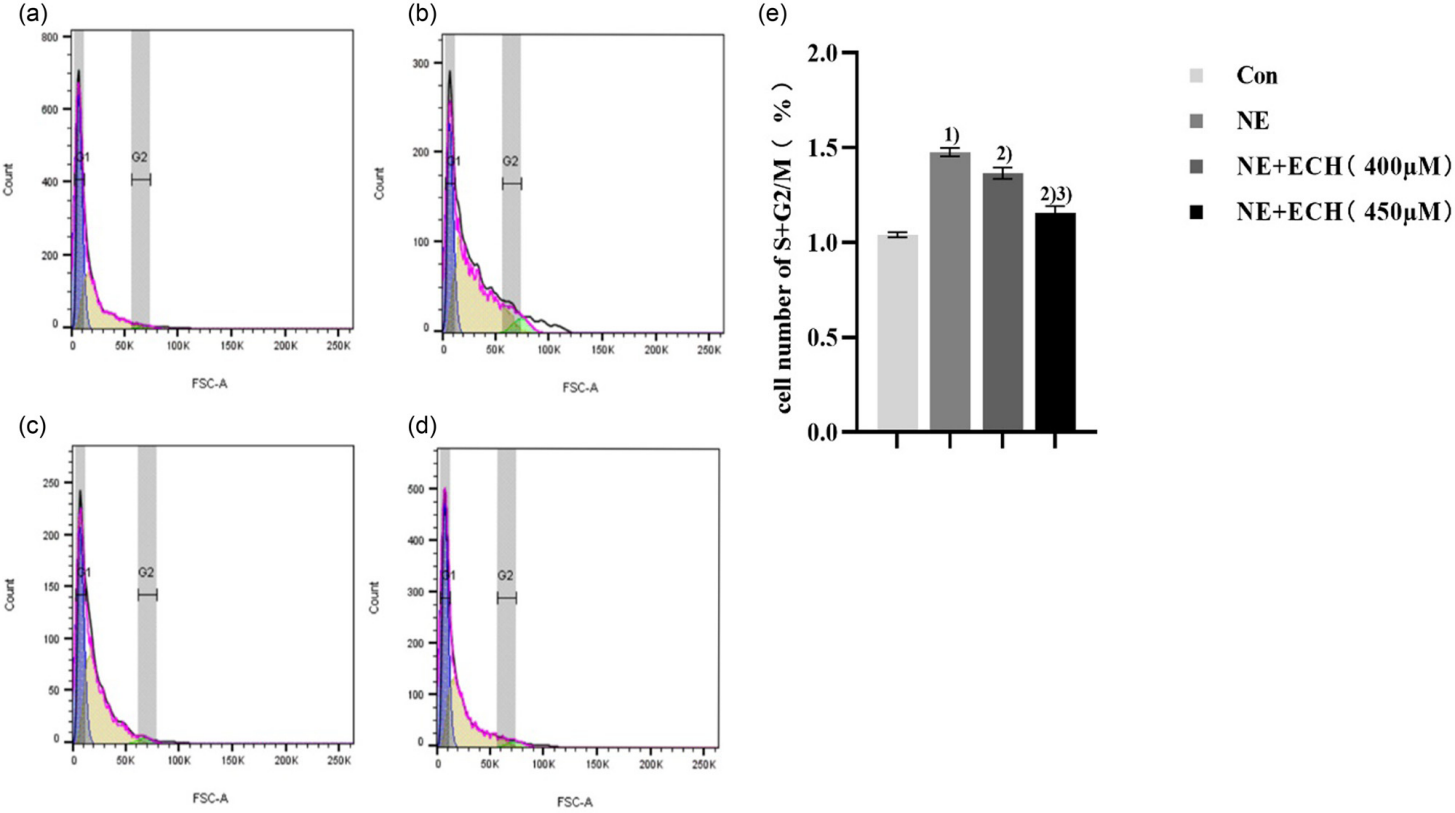
Flow cytometry was employed to identify the cycles of PASMCs. (a) Control group; (b) NE group; (c) NE + ECH (400 μM) group; (d) NE + ECH (450 μM) group and (e) cell number of S+G2/M (%) here. ^1)^ *p* < 0.01 vs control group; ^2)^ *p* < 0.01 vs NE group; ^3)^ *p* < 0.01 vs NE + ECH (400 μM) group. “Con” stands for “control.”

A significant increase in the NE group’s cell count at the S + G2/M phases in comparison to the control group is shown by the flow cytometry data in Figure 6 (*p* < 0.05). In comparison to the NE group, the NE + ECH (400 μM) group exhibited a substantially reduced amount of cells at the S + G2/M phase (*p* < 0.01). Implementing ECH (400 μM) as a pretreatment demonstrated a strong inhibitory effect on cell cycle progression, specifically at the S + G2/M phase. Furthermore, upon comparing the NE + ECH (400 μM) group with the NE + ECH (450 μM) group, a significant decrease in the number of cells in the S + G2/M phase was observed (*p* < 0.05). The results indicate that ECH suppresses the NE-induced proliferation of PASMCs in a concentration-dependent manner, primarily during the S and G2/M phase.

### 3.5 The influence of ECH on calcium overload in PASMCs caused by NE

**Figure 7:**
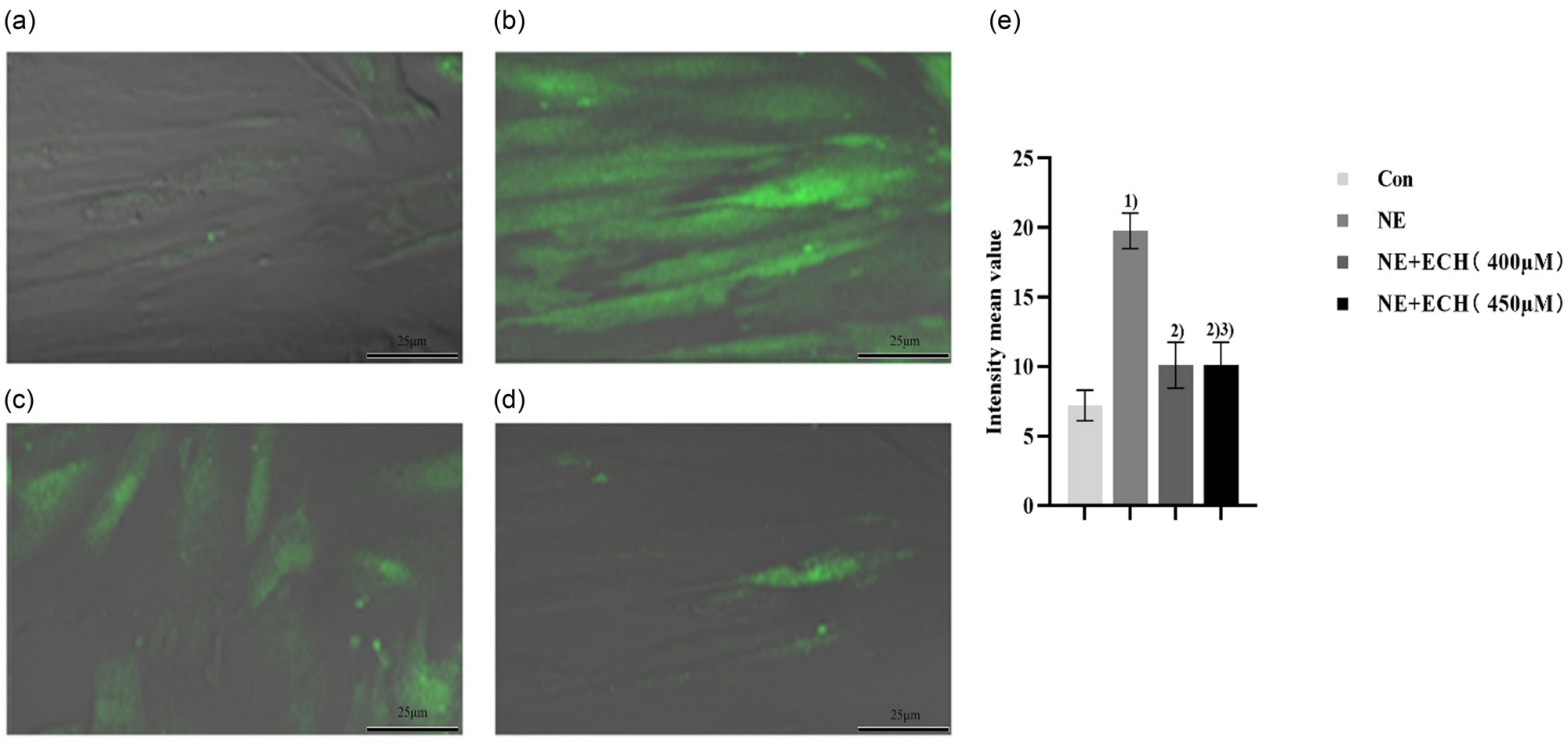
ECH’s impact on PASMCs’ Ca^2+^ excess caused by NE. (a) Control group (×600); (b) NE group (×600); (c) NE + ECH (400 μM) group (×600); and (d) NE + ECH (450 μM) group (×600). (e) Impact of ECH on NE-induced calcium excess in PASMCs, a quantitative representation of the mean fluorescence intensity. ^1)^ *p* < 0.01 vs control group; ^2)^ *p* < 0.01 vs NE group; ^3)^ *p* < 0.01 vs NE + ECH (400 μM) group. “Con” means control.

**Figure 8:**
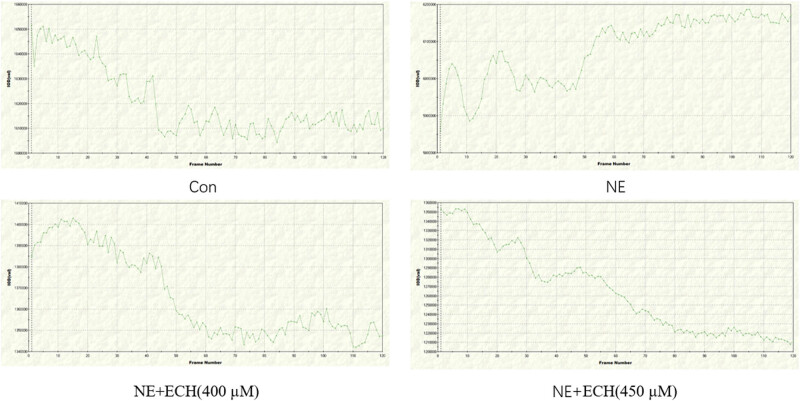
The influence of ECH on the overproduction of Ca^2+^ in PASMCs caused by NE. PASMC fluorescence intensity changes were tracked in real time. “Con” stands for “control”.

According to the data presented in Figure 7, it is evident that the NE group revealed a substantial increase in the fluorescence intensity of PASMCs than the control group (*p* < 0.01). When compared to the NE group, the NE + ECH (400 μM) group exhibited a notable reduction in fluorescence intensity, with statistical significance (*p* < 0.01). Real-time observation of the changes in intracellular fluorescence intensity in Figure 8 shows that the fluorescence intensity in the control group gradually decreased with time, proving that prolonged laser irradiation may cause fluorescence quenching. However, the fluorescence intensity in the NE group gradually increased with time, demonstrating that NE induces an increase in Ca^2+^ levels in PASMCs. By contrast, the intracellular fluorescence intensity in the NE + ECH (400 μM) group gradually decreased. The intracellular fluorescence intensity in the NE + ECH (450 μM) group was markedly reduced and maintained at a plateau level compared to the NE + ECH (400 μM) group, proving the inhibitory activities of NE + ECH (400 μM and 450 μM) groups by inhibiting the NE-induced increased [Ca^2+^]_cyt_ of PASMCs in a concentration-dependent manner.

### 3.6 Effect of ECH on internal Ca^2+^ release and external Ca^2+^ influx in PASMCs

**Figure 9:**
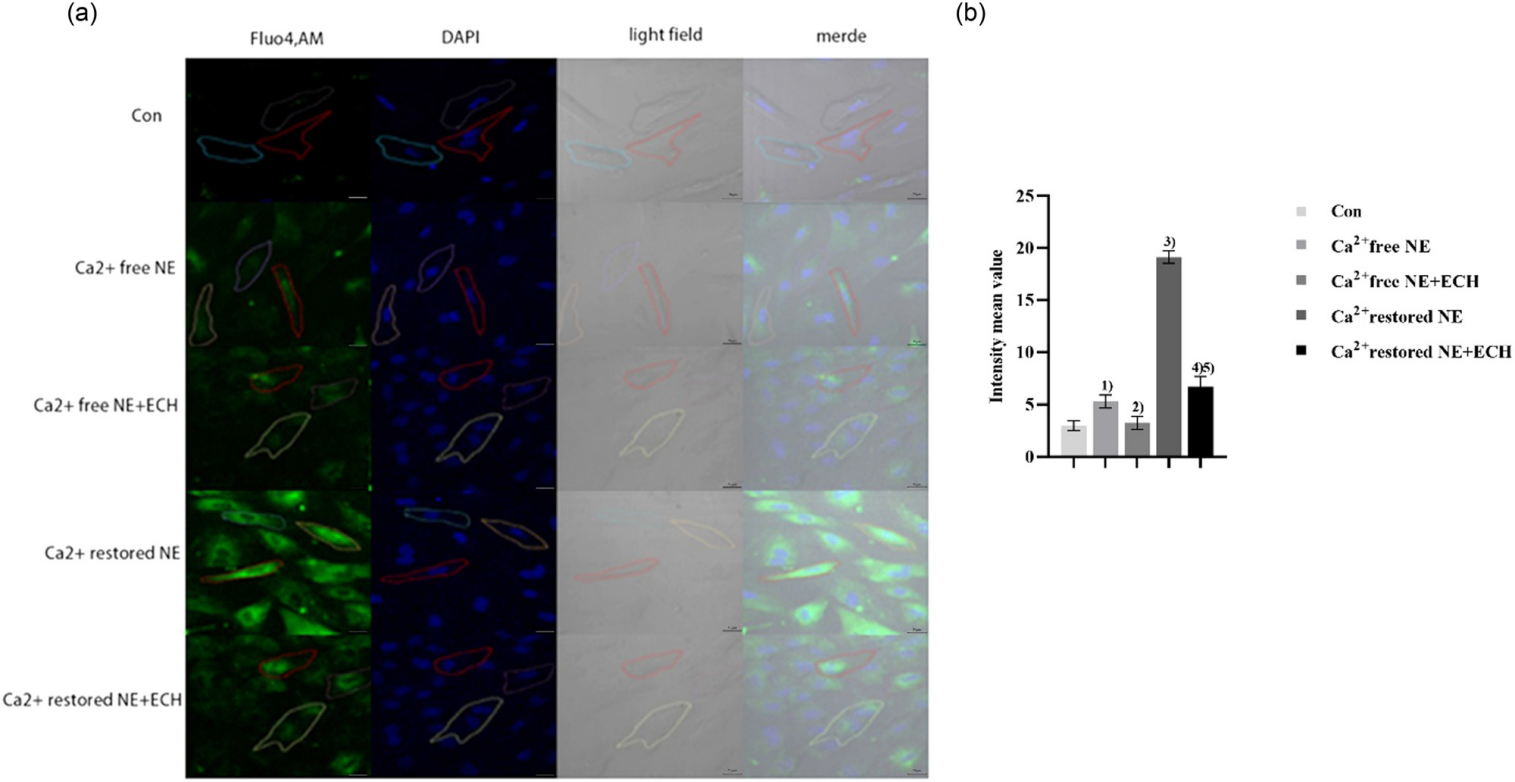
Effect of ECH on Ca^2+^ release and Ca^2+^ influx in PASMCs. (a) Fluorescence peak; (b) peak intensity averaged quantitative images. ^1)^ *p* < 0.01 vs control group; ^2)^ *p* < 0.05 vs Ca^2+^-free NE group; ^3)^ *p* < 0.01 vs Ca^2+^ -free NE group; ^4)^ *p* < 0.01 vs Ca^2+^ recovered NE group; ^5)^ *p* < 0.01 vs Ca^2+^-free NE + ECH group. “Con” means control.

According to the data presented in Figure 9, the level of intracellular fluorescence in the group with no calcium ions (Ca^2+^-free NE group) was found to be considerably greater compared to the control group (*p* < 0.01). Contrary to the Ca^2+^-free NE group, the fluorescence intensity in the Ca^2+^-free NE + ECH group exhibited a substantial drop (*p* < 0.05). NE stimulated the release of intracellular calcium ions from PASMCs. Conversely, ECH prevented the release of Ca^2+^ from intracellular Ca^2+^ stores. This was demonstrated by the observation that restoring extracellular Ca^2+^ concentration in the NE group increased intracellular Ca^2+^ concentration.

Furthermore, the fluorescence intensity of intracellular Ca^2+^ was much higher when extracellular Ca^2+^ was restored than when it was not restored (*p* < 0.01). The fluorescence intensity of the NE + ECH group with recovered Ca^2+^ was lower compared to the NE group with recovered Ca^2+^ (*p* < 0.01). ECH was proven to inhibit Ca^2+^ influx from the outside of PASMCs.

**Figure 10:**
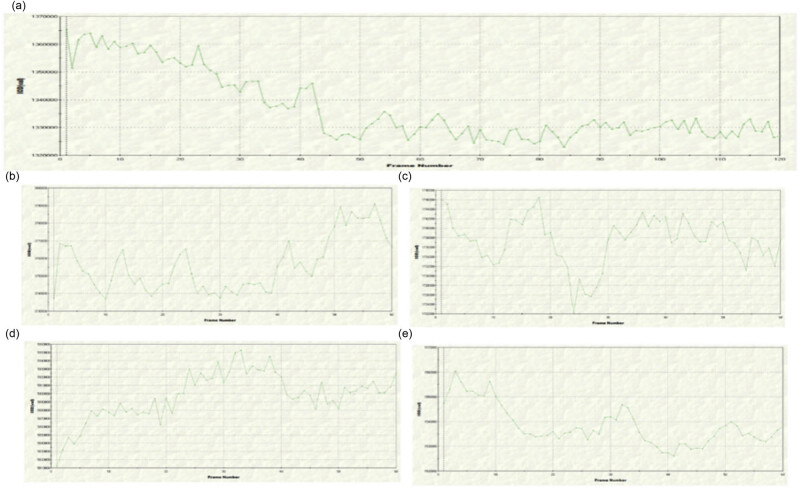
Effect of ECH on internal Ca^2+^ release and external Ca^2+^ influx in PASMCs changes in fluorescence intensity of PASMCs were monitored in real time. (a) Control group; (b) Ca^2+^ free NE group; (c) Ca^2+^ free NE + ECH group; (d) Ca^2+^ restored NE group; (e) Ca^2+^ restored NE + ECH group. Con” means control.

Figure 10 shows the change curve of fluorescence intensity in PASMCs monitored in real time. In the control group, the fluorescence intensity in PASMCs gradually decreases, proving that there is fluorescence quenching after long-time laser irradiation. The real-time fluorescence curve of the Ca^2+^-free NE group showed that the intracellular fluorescence fluctuated regularly at the beginning and then increased gradually with time, proving that NE could induce intracellular Ca^2+^ release and increase the level of intracellular Ca^2+^. The real-time fluorescence intensity curve of the Ca^2+^-free NE + ECH group showed that the intracellular fluorescence intensity decreased gradually, which proved that the ECH preincubated PASMCs could inhibit the release of internal Ca^2+^ in PASMCs induced by NE. The real-time fluorescence curve of the Ca^2+^ restored NE group showed that after the recovery of extracellular Ca^2+^, the intracellular fluorescence intensity increased rapidly, which proved that NE could induce the extracellular Ca^2+^ influx of PASMCs. The real-time fluorescence curve of the Ca^2+^ restored NE + ECH group revealed that the intracellular fluorescence intensity decreased slowly with time, which proved that ECH could inhibit the extracellular Ca^2+^ influx induced by NE.

### 3.7 ECH decreased mPAP and RVHI in rats

**Figure 11:**
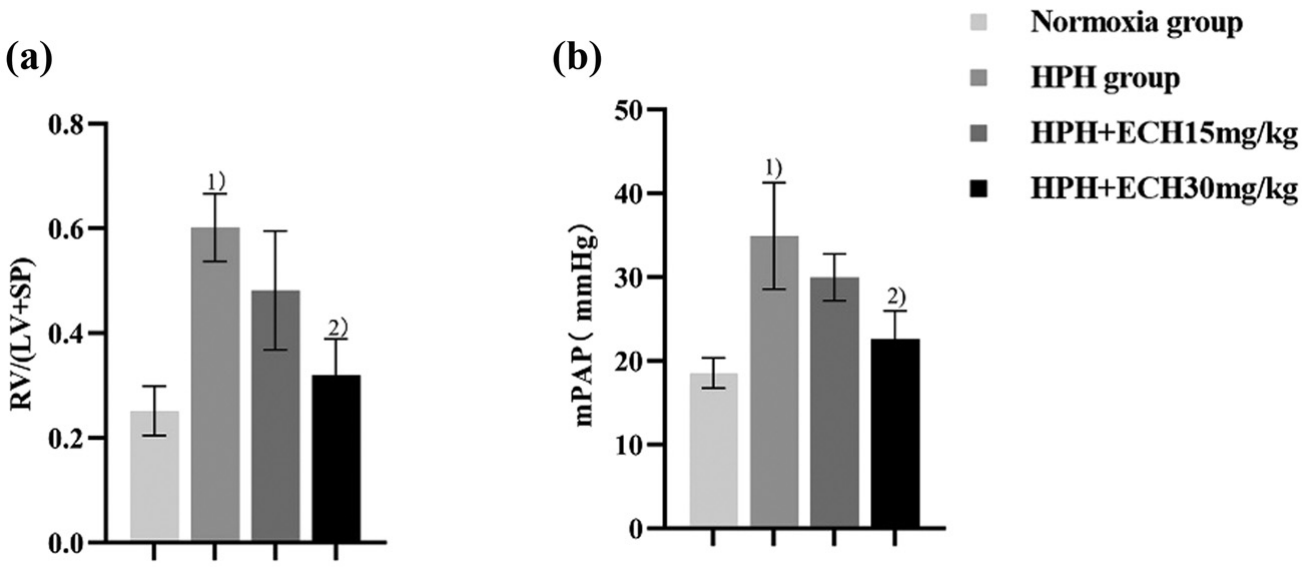
Effects of ECH on pulmonary artery pressure and RVHI in HPH rats. (a) RVHI and (b) mPAP. ^1)^ *p* < 0.05 vs normoxic group; ^2)^ *p* < 0.05 vs HPH group. (*n* = 6).

According to the data presented in Figure 11, the mPAP and RVHI of rats in the normoxia group were measured to be 18.54 ± 1.81 mmHg and 0.25 ± 0.05, respectively. On the other hand, the mPAP and RVHI of rats in the HPH group were observed to be 34.91 ± 6.36 mmHg and 0.60 ± 0.06, respectively. The rats in the HPH group demonstrated considerably elevated mPAP and RVHI than the normoxia group (all *p* < 0.05), suggesting successful rat modeling. The mPAP and RVHI of rats in the treatment group exhibited a decrease. Specifically, the mPAP and RVHI values for rats in the ECH (15 mg/kg) group were recorded as 29.98 ± 2.81 mmHg and 0.48 ± 0.1, respectively. Similarly, the mPAP and RVHI values for rats in the HPH + ECH (30 mg/kg) group were 22.60 ± 3.40 mmHg and 0.32 ± 0.07, respectively. In comparison to the HPH group, both mPAP and RVHI demonstrated a substantial drop (*p* < 0.05). Notably, the reduction in mPAP and RVHI was more pronounced in the ECH (30 mg/kg) group, proving that ECH can mitigate pulmonary artery pressure and right heart hypertrophy in rats.

### 3.8 ECH improves pulmonary artery remodeling in rats

**Figure 12:**
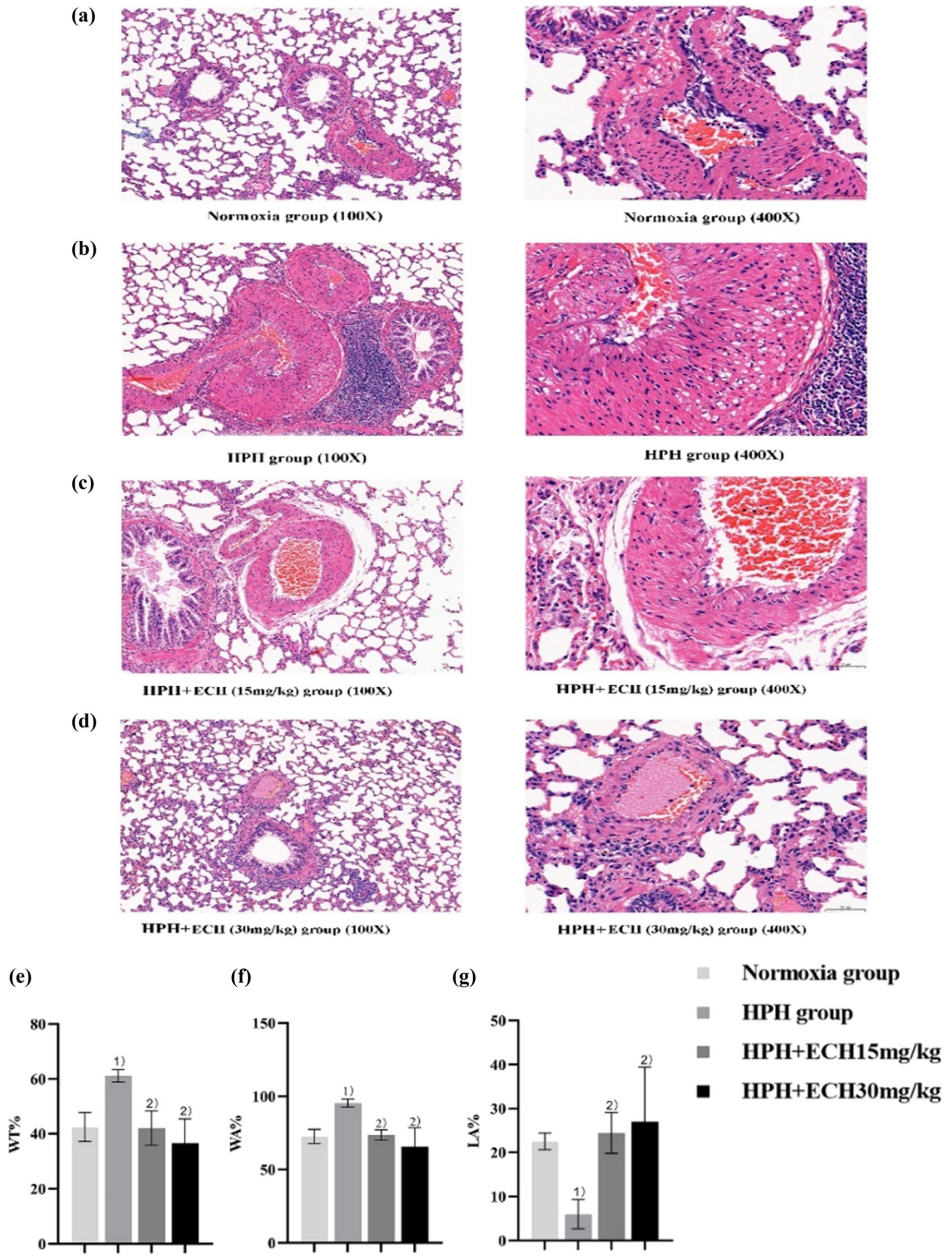

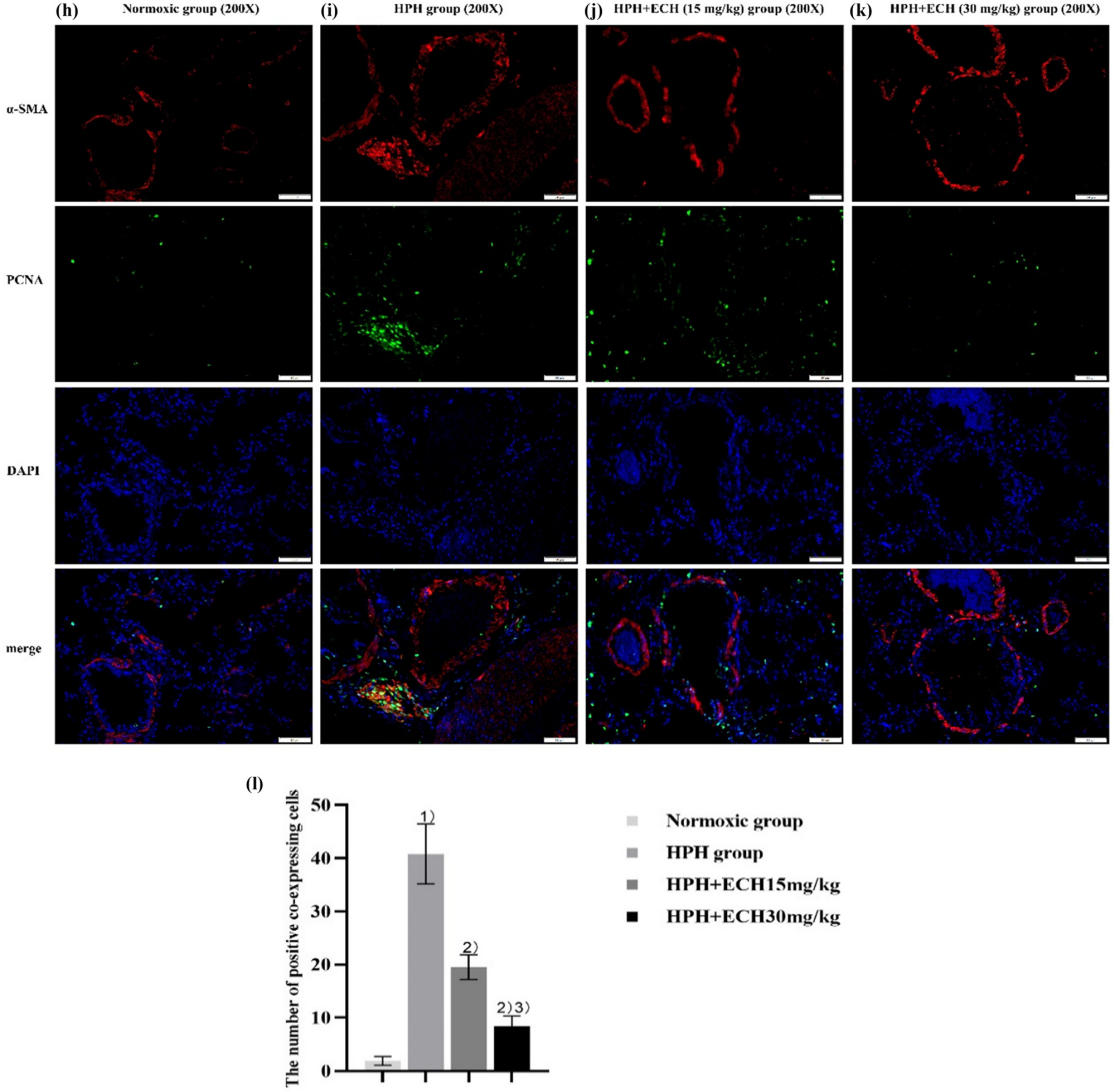
Effect of ECH on pulmonary artery remodeling in HPH rats. (a)–(d) HE staining results of lung tissue of rats in each group, (e) WT%, (f) pulmonary vascular lumen area/total wall area (WA%), (g) pulmonary vascular lumen area/total wall area (LA%), and (h)–(k) results of immunofluorescence staining of lung tissue in each group, DAPI stained the nucleus blue; PCNA showed positive expression green; mainly expressed in the nucleus. The positive expression of α-SMA is red and is mainly expressed in the cytoplasm. (l) Number of positive co-expressing cells stained by immunofluorescence. ^1)^ *p* < 0.05 vs normoxic group; ^2)^ *p* < 0.05 vs HPH group; ^3)^ *p* < 0.05 vs HPH + ECH (15 mg/kg) group (*n* = 6).

The histopathological examination of lung tissue samples from each experimental group revealed that the pulmonary artery wall of rats in the normoxic group exhibited a smooth appearance, devoid of any noticeable infiltration of inflammatory cells or excessive proliferation of fibrous tissue. Furthermore, the cells were observed to be organized in a well-structured and compact manner. In the HPH group, there was a notable increase in the thickness of the pulmonary artery wall in rats. In addition, the lumen of the pulmonary artery exhibited a considerable narrowing, accompanied by a disorganized arrangement of cells. The therapy group showed reduced pulmonary artery wall thickening and a more organized arrangement of cells, as depicted in Figure 12(a), (d), (e), and (g). The data about WT%, WA%, and LA% were synthesized by examining the pulmonary artery in each group of rats. The WT%, WA%, and LA% of rats in the normoxic group were recorded as follows: The values for the experimental group were 42.46 ± 5.30, 72.56 ± 4.80, and 22.52 ± 1.86, while the rats in the normoxic group had values of 61.11 ± 2.23, 95.45 ± 2.68, and 5.98 ± 33.4. WT% and WA% exhibited a substantial rise compared to the normoxic group, while the LA% exhibited a considerable decline (*p* < 0.05). In the HPH + ECH (15 mg/kg) group, the WT%, WA%, and LA% were measured to be 42.06 ± 6.22, 73.45 ± 3.51, and 24.45 ± 4.64, respectively.

Similarly, in the HPH + ECH (30 mg/kg) group, the WT%, WA%, and LA% were found to be 36.60 ± 8.85, 65.40 ± 13.04, and 26.92 ± 15.52, respectively. In comparison to the HPH group, the WT% and WA% exhibited a statistically significant drop, while the LA% demonstrated a statistically significant increase in both experimental groups.

Figure 12(h)–(l) shows the staining results of immunofluorescence of rats in each group. The fluorescence expression of α-SMA and PCNA in the normoxic group was the weakest, compared with normoxic group, the fluorescence expressions of α-SMA and PCNA in HPH group were significantly increased (*p* < 0.05). Compared with HPH, the fluorescence expressions of α-SMA and PCNA in the treatment group were significantly decreased (all *p* < 0.05).

The findings of our work revealed that the administration of ECH may have a beneficial effect on the remodeling of the pulmonary artery in rats with HPH.

### 3.9 Identification of TRPC1/6 and CaM proteins by western blotting

**Figure 13:**
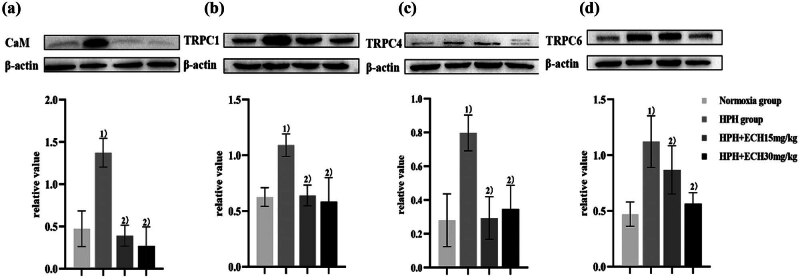
*In vivo* experiment western blot analysis of TRPC1/4/6 and CaM proteins. (a) CaM protein expression in rat lung tissue; (b) TRPC1 protein expression in rat lung tissue; (c) TRPC4 protein expression in rat lung tissue; and (d) TRPC6 protein expression in rat lung tissue. ^1)^ *p* < 0.05 vs normoxic group; ^2)^ *p* < 0.05 vs HPH group. (*n* = 3).

The results of *in vivo* tests are depicted in Figure 13a–d. TRPC1, TRPC4, and TRPC6 expressions in the HPH group of rats were considerably higher than in the normoxic group. In contrast, the levels of TRPC1, TRPC4, and TRPC6 expressions in rats from both treatment groups exhibited a considerable drop. Contrary to the normoxic group, the HPH group exhibited a substantial rise in CaM expression, whereas the treatment group demonstrated a significant decrease in CaM expression (all *P* < 0.05).

## 4 Discussion

In this study, both *in vitro* and *in vivo* experiments were conducted to elucidate the mechanisms underlying ECH’s efficacy in treating HPH. Current research indicates that hypoxia stimulates sympathetic nerve activity, leading to increased production of norepinephrine and epinephrine. This surge results in an excessive influx of Ca^2+^ into PASMCs, causing calcium overload and abnormal PASMCs proliferation, a process closely linked to PVR [13,14]. To simulate this, an *in vitro* model using NE-induced PASMCs was developed to replicate calcium overload and abnormal proliferation under hypoxic conditions. Flow cytometry was used to determine the specific phase in the PASMC proliferation cycle where ECH exerts its inhibitory effect. The findings revealed that ECH impedes PASMC proliferation by inhibiting the cell cycle at the S + G2/M phase. Furthermore, to assess changes in intracellular Ca^2+^ concentration, Fluo-4 AM was employed to measure [Ca^2+^]_cyt_ in PASMCs. Results indicated that ECH effectively inhibits both Ca^2+^ release and inflow, suggesting its primary action on Ca^2+^ channels in the PASMC membrane.

This study aimed to investigate Ca^2+^-related channels on PASMC membranes. It is well documented that nearly all TRPC protein family members are activated when intracellular calcium stores in the endoplasmic reticulum–sarcoplasmic reticulum are depleted,^7^ highlighting TRPC’s crucial role in intracellular Ca^2+^ regulation. Research indicates that SOCC is significant in regulating intracellular [Ca^2+^]_cyt_, with SOCC inhibition leading to a reduction in store-operated calcium entry (SOCE) and consequently decreasing [Ca^2+^]_cyt_ [15]. TRPC1 and TRPC4 are identified as primary components of SOCC [16,17]. TRPC, being a Ca^2+^ channel on the plasma membrane, is also essential for ROCC formation. Specifically, TRPC6 is reported to constitute ROCC in PASMCs [18], underlining the TRPC family’s involvement in pulmonary vascular function regulation. TRPC1, TRPC4, and TRPC6 are notably studied in the context of HPH [19]. Moreover, previous findings suggest that ECH can relax pulmonary blood vessels during NE-induced pulmonary vasoconstriction, indicating that ECH’s inhibitory effect on NE-induced PASMC contraction might be mediated through Ca^2+^-dependent signaling pathways. CaM is a key regulator of PASMC contraction. An increase in [Ca^2+^]_cyt_ activates CaM, leading to the formation of Ca^2+^/CaM complexes and inducing contraction of the smooth muscle layer. Therefore, this study concentrates on exploring the effects of ECH on TRPC1, TRPC4, TRPC6, and CaM *in vivo* settings.

The experimental outcomes revealed that *in vivo* studies indicated an increase in CaM expression in the lung tissues of HPH rats, which was significantly reduced following treatment with ECH at 15 and 30 mg/kg dosages. This intervention led to the alleviation of PVR and a reduction in mPAP in HPH rats. These findings suggest that ECH may mitigate Ca^2+^ overload in PASMCs by attenuating the overexpression of TRPC1, TRPC4, TRPC6, and CaM, thereby reducing PVR and mPAP and potentially preventing HPH.

We believe that ECH has a strong potential in treating HPH, and at present, the drugs on the market for the treatment of HPH are mainly aimed at dilating pulmonary blood vessels to alleviate the development of HPH; however, drugs for the treatment of HPH-induced PVR have yet to be developed. Through the current research of this research group, it is found that ECH can not only diastolic abnormal pulmonary vasoconstriction in NE-induced rats but also improve pulmonary artery remodeling in HPH rats, and therefore, ECH deserves our further study.

However, the present study only studied the effects of ECH on several important proteins related to ROCC, SOCC, and CaM. Future studies should aim to expand the scope of detection and investigate the effects of ECH on other proteins involved in regulating Ca^2+^ channels in PASMCs. In addition, it is critical to explore the effects of ECH on various indicators of downstream pathways associated with the growth and contraction of PASMCs *in vitro*. Future studies might employ techniques such as the patch-clamp method to study the influence of ECH on additional Ca^2+^ channels in PASMCs. These extended research efforts are vital for a more comprehensive understanding of ECH’s therapeutic mechanisms in HPH.

## 5 Conclusion

In summary, the findings of this investigation demonstrate that ECH exerts its effects through the TRPC1, TRPC4, and TRPC6 and CaM signaling pathways. These effects include the inhibition of contraction and proliferation of PASMCs, reduction of mPAP in rats with HPH, attenuation of pulmonary remodeling in HPH rats, and mitigation of RVHI.
